# Supplementary material for: A systematically-revised ribosome profiling method for bacteria reveals pauses at single-codon resolution
Source: eLife. 2019 Feb 6;8:e42591. doi: 10.7554/eLife.42591 (PMC6377232; doi:10.7554/eLife.42591)
Supplement: Figure 2—source data 1. [file elife-42591-fig2-data1.pdf]

Library	Sample_#	Media Treatment	Lysis Buffer	Harvest	Reference	SRA accession number
L1	CW63	Cm	Cm	Filter	This paper	Pending
L2	OL38	Cm	Cm	Filter	Marks, Mankin, PNAS 2016	SRR4190324
L3	OL34	Cm	Cm	Centrifuge	Latif, Palsson, BioTechniques 2015	SRR1693437
L4	OL35	Cm	Cm	Centrifuge	Latif, Palsson, BioTechniques 2015	SRR1693438
L5	OL18	Cm	Cm	Centrifuge	Liu, Roberts, PNAS 2013	SRR869826
L6	OL19	Cm	Cm	Centrifuge	Liu, Roberts, PNAS 2013	SRR869827
L7	OL20	Cm	Cm	Centrifuge	Liu, Roberts, PNAS 2013	SRR869828
L8	OL21	Cm	Cm	Centrifuge	Liu, Roberts, PNAS 2013	SRR869829
L9	OL1	Cm	Cm	Centrifuge	Oh, Bukau, Cell 2011	SRR364364
L10	OL2	Cm	Cm	Centrifuge	Oh, Bukau, Cell 2011	SRR364366
L11	OL24	none	Cm	Filter	Baggett, Gross, PLOS Genetics 2017	SRR4421280
L12	OL25	none	Cm	Filter	Baggett, Gross, PLOS Genetics 2017	SRR4421281
L13	OL26	none	Cm	Filter	Baggett, Gross, PLOS Genetics 2017	SRR4421284
L14	OL27	none	Cm	Filter	Burkhardt, Gross, eLife 2017	SRR3147100
L15	OL28	none	Cm	Filter	Burkhardt, Gross, eLife 2017	SRR5186136
L16	OL29	none	Cm	Filter	Burkhardt, Gross, eLife 2017	SRR5186137
L17	OL30	none	Cm	Filter	Burkhardt, Gross, eLife 2017	SRR5186138
L18	OL12	none	Cm	Filter	Li, Weissman, Nature 2012	SRR407274-5 combined
L19	OL13	none	Cm	Filter	Li, Weissman, Nature 2012	SRR407276-7 combined
L20	OL14	none	Cm	Filter	Li, Weissman, Cell 2014	SRR1067765-8 combined
L21	OL36	none	Cm	Filter	Marks, Mankin, PNAS 2016	SRR4190326
L22	OL10	none	Cm	Filter	Haft, Landick, PNAS 2014	SRR1211047
L23	OL11	none	Cm	Filter	Haft, Landick, PNAS 2014	SRR1211048
L24	OL16	none	Cm	Filter	Subramaniam, O'Shea, Cell 2014	SRR1301057
L25	OL17	none	Cm	Filter	Subramaniam, O'Shea, Cell 2014	SRR1301059
L26	CW60	none	Cm	Filter	Mohammad, Buskirk, Cell Rep 2016	SRR2340141
L27	FM22	none	150 mM MgCl2	Filter	This Paper	Pending
L28	FM20	none	1 M NaCl	Filter	This Paper	Pending
L29	FM85	none	150 mM MgCl2	Filter	This Paper	Pending
L30	FM87	MPC	150 mM MgCl2	Filter	This Paper	Pending
L31	FM88	none	Cm	Filter	This Paper	Pending
L32	FM90	MPC	Cm	Filter	This Paper	Pending
L33	FM91	none	150 mM MgCl2	Direct Freeze	This Paper	Pending
L34	FM93	MPC	150 mM MgCl2	Direct Freeze	This Paper	Pending
L35	FM67	none	150 mM MgCl2	Direct Freeze	This Paper	Pending
